# Crosstalk of Genetic Variants, Allele-Specific DNA Methylation, and Environmental Factors for Complex Disease Risk

**DOI:** 10.3389/fgene.2018.00695

**Published:** 2019-01-09

**Authors:** Huishan Wang, Dan Lou, Zhibin Wang

**Affiliations:** ^1^Laboratory of Human Environmental Epigenome, Department of Environmental Health and Engineering, Bloomberg School of Public Health, Johns Hopkins University, Baltimore, MD, United States; ^2^Li Ka Shing Faculty of Medicine, The University of Hong Kong, Pokfulam, Hong Kong; ^3^State Key Laboratory of Biocatalysis and Enzyme Engineering, School of Life Sciences, Hubei University, Wuhan, China

**Keywords:** allele-specific DNA methylation (ASM), single nucleotide polymorphisms (SNPs), allele-specific gene expression (ASE), genetic variants, regional “autosomal chromosome inactivation (ACI)”, quantitative trait locus (QTL), allele-specific binding of transcription factors (ABTFs), SNP intensifier model

## Abstract

Over the past decades, genome-wide association studies (GWAS) have identified thousands of phenotype-associated DNA sequence variants for potential explanations of inter-individual phenotypic differences and disease susceptibility. However, it remains a challenge for translating the associations into causative mechanisms for complex diseases, partially due to the involved variants in the noncoding regions and the inconvenience of functional studies in human population samples. So far, accumulating evidence has suggested a complex crosstalk among genetic variants, allele-specific binding of transcription factors (ABTF), and allele-specific DNA methylation patterns (ASM), as well as environmental factors for disease risk. This review aims to summarize the current studies regarding the interactions of the aforementioned factors with a focus on epigenetic insights. We present two scenarios of single nucleotide polymorphisms (SNPs) in coding regions and non-coding regions for disease risk, via potentially impacting epigenetic patterns. While a SNP in a coding region may confer disease risk via altering protein functions, a SNP in non-coding region may cause diseases, via SNP-altering ABTF, ASM, and allele-specific gene expression (ASE). The allelic increases or decreases of gene expression are key for disease risk during development. Such ASE can be achieved via either a “SNP-introduced ABTF to ASM” or a “SNP-introduced ASM to ABTF.” Together with our additional in-depth review on insulator CTCF, we are convinced to propose a working model that the small effect of a SNP acts through altered ABTF and/or ASM, for ASE and eventual disease outcome (named as a “SNP intensifier” model). In summary, the significance of complex crosstalk among genetic factors, epigenetic patterns, and environmental factors requires further investigations for disease susceptibility.

## Introduction

Genetic variants identified from genome-wide association studies (GWAS) promise to uncover the understanding of inter-individual differences in phenotypes and the risk of complex diseases. One hypothesis is that the susceptibility of an individual to a complex disease is due to the interaction of genetic variants with environmental factors acting through epigenetic mechanisms. Therefore, understanding the complex crosstalk among genetic variation, environmental exposure, and epigenetic patterns is essential for unraveling the etiology of common disease. This review aims to illustrate potential mechanisms for the crosstalk of genetic factors, epigenetic patterns, and allelic binding of transcriptional factors (ABTF), as well as the crosstalk with environmental exposure for disease susceptibility. We begin the review with a basic and brief, but hopefully necessary introduction on the fundamental insights of epigenetic mechanisms (DNA methylation and histone modifications) in the regulation of gene transcription, because both DNA methylation and histone modifications are linked with genetic variants and environmental exposure for disease or complex traits in many burgeoning reports (detailed below). We then describe the crosstalk among different factors (including genetic variation, epigenetic variation, gene expression, and environmental factors) for complex disease risk. We complement the aforementioned broad topics with an in-depth summary on the crosstalk among insulator CTCF, genetic variation, and epigenetic variation in disease or complex traits. This subtopic is currently of high interest in the biomedical arena because of CTCF's pleiotropic roles in biology. CTCF helps shape the high-order genome organization (e.g., “4D nucleosome” program), acts as a tumor suppressor (Kemp et al., 2014), and plays an important role in complex traits. To better envision the underlying mechanisms, we selectively discuss the most relevant investigations regardless of the disease investigated. That is, we did not focus on one type of complex diseases, because a relevant functional study may not be available if we restrain our references to one disease. Hence, with such selection, we may unintentionally overlook many important reports within a given disease. In cases where the expected or hypothesized mechanism may not have been thoroughly addressed, we try to provide a diagram to aid our illustration. We will start our review with brief information on epigenetic background.

## Epigenetic Mechanisms Involved in Transcriptional Regulation

### DNA Methylation Patterns: Establishment, Maintenance, and Functional Roles

DNA methylation patterns are established and maintained by three DNA methyltransferases (Dnmts), namely Dnmt1, Dnmt3a, and Dnmt3b, in both the mouse and human genomes (Bestor, 2000). In a simplified “two-step” model, it is the role of Dnmt3a and Dnmt3b, which initiate methylation of globally hypomethylated genomic DNA (after implantation) to establish methylation patterns during early embryonic development (Okano et al., 1999). Afterwards, via coupling with DNA replication machinery, Dnmt1 faithfully copies methylation information from the parental strands to the daughter strands during DNA replication (Jones and Liang, 2009; Jurkowska et al., 2011). Because of this copying role, Dnmt1 is referred to as the “maintenance methyltransferase,” whereas Dnmt3a and Dnmt3b are called the “de novo methyltransferases.” In support of this model, Dnmt3a and Dnmt3b have high activity toward unmethylated DNA, while Dnmt1 shows low activity toward unmethylated DNA and prefers hemi-methylated DNA (Jeltsch, 2006; Jurkowska et al., 2011).

**Figure 1:**
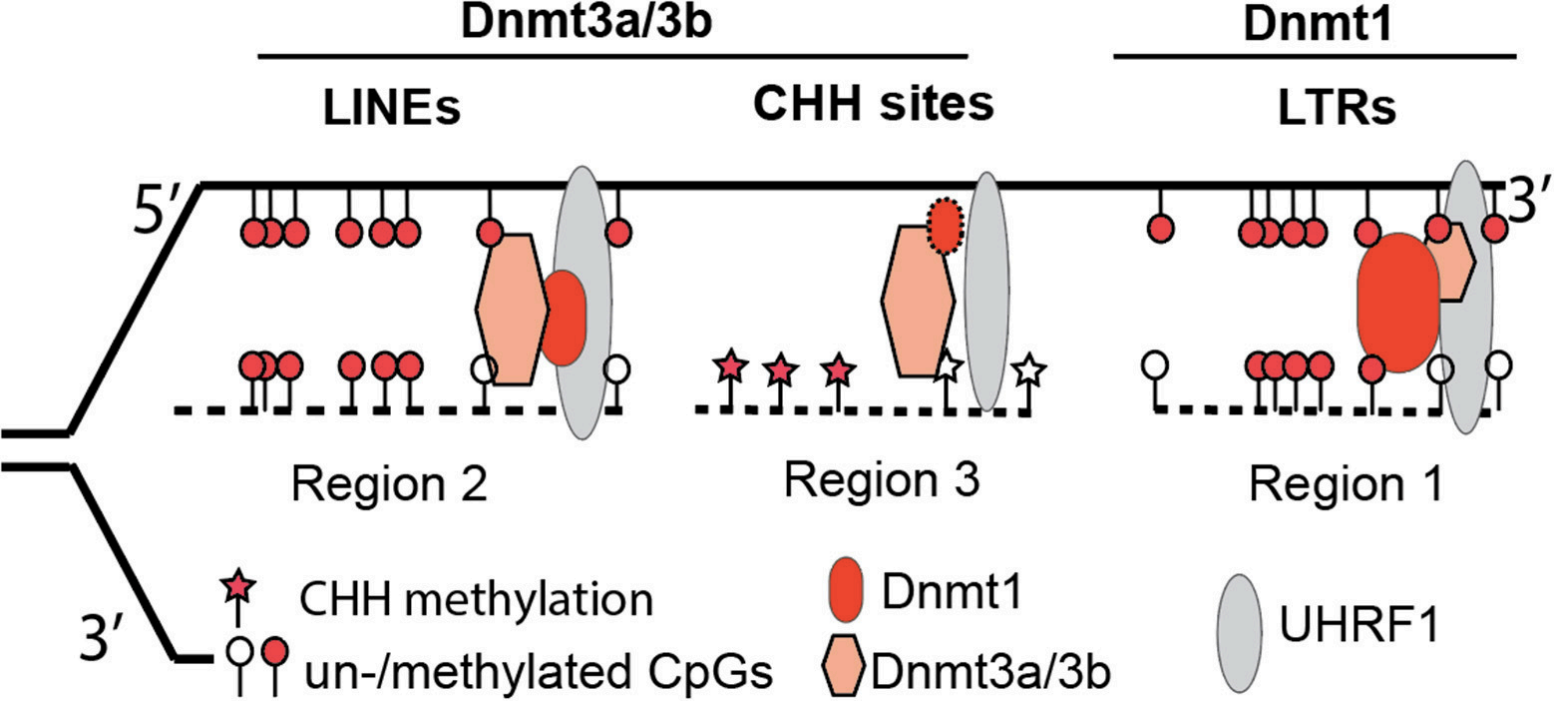
A revised model illustrating the complementary and concerted actions from Dnmt3a/3b *de novo* activity and Dnmt1 maintenance activity at three representative genomic regions. Via interacting with proteins such as UHRF1 at replication forks, Dnmt1 plays a dominant role in retaining methylation at retrotransposon LTRs (region 1), whereas Dnmt3a/3b are mainly responsible for methylation at retrotransposon LINEs (region 2) or genomic regions with CHH (H = A, T, or C) sites (region 3). The loss of Dnmt3a/3b or Dnmt1 results in hypomethylated retrotransposon LTRs or LINEs. The size of each symbol represents the degree of importance for protein factors in maintaining methylation patterns in particular genomic regions, with the bigger symbol being the more important in maintenance.

However, with the recent availability of base-resolution methylome data for Dnmt knockout cells, this “two-step” model seems oversimplified (Li et al., 2015). Our data demonstrate that in the absence of Dnmt1, Dnmt3a and Dnmt3b can still maintain symmetrical methylation of CpG dinucleotides (where a cytosine nucleotide is followed by a guanine nucleotide linearly along 5′ to 3′ direction) to some degree. In addition, the presence of Dnmt1 alone allows cells to maintain the methylation levels of retrotransposon long terminal repeats (LTRs) in Dnmt double knockout cells (*Dnmt3a*^−/−^/*3b*^−/−^), at levels comparable to wild type cells. In *Dnmt1*^−/−^, the presence of only two *de novo* methyltransferases (Dnmt3a and Dnmt3b) still enables embryonic stem cells to retain methylation at retrotransposon long interspersed nuclear elements (LINEs). Therefore, Dnmt1 is required for methylation of LTRs, whereas Dnmt3a and Dnmt3b are necessary for LINE methylation (Figure 1) (Li et al., 2015). Collectively, it is more accurate to state that the concerted actions of three Dnmt enzymes are required for the maintenance and establishment of DNA methylation patterns in the mouse genome. While at the last stage of publishing this review, a new report suggests that Dnmt1 does have de novo methyltransferase activity (Li et al., 2018).

The unexpected division of labor between maintenance Dnmt1 and *de novo* Dnmt3a and Dnmt3b for retrotransposons is informative for deeper mechanistic insights in future investigations. In early epidemiological studies, the methylation levels of LINEs or Alu sequences in the human/mouse genome were assumed to represent global DNA methylation changes. Though the assumption was largely untested (Nelson et al., 2011), LINE or Alu methylation has been used as a biomarker for environmental exposure or a diseased state. For example, in a recent cohort study that examined the association between DNA methylation of pre-diagnostic leukocyte and gastric cancer risk, it was reported that the latter was inversely associated with Alu methylation. Intriguingly, LINE methylation was not associated with gastric cancer risk (Gao et al., 2012). The association of Alu but not LINE methylation with gastric cancer risk is in line with other distinct mechanisms in control of different classes of retrotransposons (Li et al., 2015). Presumably, the mechanism for Alu methylation was affected in gastric cancer, but the mechanism for LINE methylation was not. In an effort to assess Alu (LINE not investigated) methylation from peripheral blood DNA of healthy donors and patients with alcohol use disorders, Kim and colleagues found that Alu methylation levels are significantly higher in the latter than the former (Kim et al., 2016). The underlying mechanism for the aforementioned associations, however, remains to be determined. In the agouti viable yellow (*A*^*vy*^) mouse studies, methylation of CpG sites within a LTR retrotransposon is vulnerable (hypomethylated) to environmental insults, including Bisphenol A (BPA), folate, and alcohol (Dolinoy, 2008; Kaminen-Ahola et al., 2010). Because LTR's methylation depends on Dnmt1 (Li et al., 2015), it may imply that Dnmt1-dependent methylation activity is more sensitive to environmental exposure. It is also worth mentioning that Dnmt1 is highly expressed in most somatic tissues, whereas Dnmt3a and Dnmt3b are not. In summary, depending on exposure-alteration of Dnmt1 or Dnmt3a/3b, the LINE or LTR methylation level should be selected accordingly, in order to represent global DNA methylation changes in cells.

Methylation of CpG sites can occur in the transcribed regions of genes (either silenced or expressed) (Lister et al., 2009), suggesting that the alteration of DNA methylation patterns may not affect the expression of a large number of genes. Indeed, recent investigations demonstrate that global DNA hypomethylation did not alter the transcriptome as drastically as previously expected (Blattler et al., 2014; Li et al., 2015). In contrast to methylation changes in a global fashion in *Dnmt*-knockout cells, environmental exposure including BPA exposure seems to change methylation patterns only at specific genomic loci (Dolinoy et al., 2007; Kundakovic et al., 2015). Therefore, if the environmental exposure-altered methylation has an important impact on gene transcription, we expect that the CpG sites bearing the altered methylation must play critical roles in gene regulation, such as CpG sites within enhancers or binding motif of critical transcriptional factors (TF) or the imprinting control regions (ICRs).

### Histone Modification for Permissive or Inhibitive Transcription

Another layer of epigenetic mechanisms for the regulation of gene expression is posttranslational modifications (PTMs) of histone tails. To date, there are a few hundred distinct histone tail modifications, including histone acetylation, methylation, and phosphorylation (Strahl and Allis, 2000; Tan et al., 2011). The former two have been known for decades to possess roles in gene transcription, and several representative histone acetylation and methylation marks have been actively pursuing by the community for many years (Allfrey et al., 1964; Barski et al., 2007; Wang et al., 2008). For example, the ENCODE project selects eight histone marks out of many potential histone acetylations and methylations: H3K4me1 (H3 lysine 4 monomethylation), H3K4me2, H3K4me3, H3K9ac (H3 lysine 9 acetylation), H3K27ac, H3K36me3, H3K9me3, and H3K27me3 (Yue et al., 2014). The former six are active histone marks in association with expressed genes, whereas the latter two are repressive marks in association with silent genes. Except H3K36me3 at gene bodies, the remaining seven histone marks can be enriched at promoters and enhancers (Dai and Wang, 2014).

Each histone mark demonstrates useful and maybe distinct information, but they can also share overlapping information (Wang et al., 2008). Seven marks can be present at promoters and/or enhancers, but each mark may better serve a particular application. H3K4me3, H3K9ac, and H3K27ac are enriched at promoters or transcription start sites of active genes (Wang et al., 2008). Similarly, H3K4me1, H3K9ac, and H3K27ac are enriched at enhancers, with H3K27ac particularly enriched at active enhancers (Creyghton et al., 2010). One active mark (out of the former five) and one inactive mark (either H3K9me3 or H3K27me3) can be used to predict bivalent promoters (Bernstein et al., 2006; Roh et al., 2006). Among potential combinations, H3K27me3 and H3K4me3 are popularly chosen for testing promoter bivalency. In addition to these popularly analyzed histone acetylations and methylations, many more new histone marks have been recently identified (Tan et al., 2011) and their roles in gene transcription and biological pathways are less clear (Goudarzi et al., 2016). Recently, even one of the well-characterized histone acetylation marks, H3K27ac, was shown to have an unexpected suppressing role. In contrast to the transcriptional activation of H3K27ac at enhancers and promoters, age-related up-regulated genes contain hyper H3K27ac in gene bodies, acting to suppress the overexpression of inflammaging genes (Cheng et al., 2018). In addition, the expression changes of these age-related genes can be predicted by gene body H3K27ac level. It seems that histone marks have the potential to reflect the aging stage or disease conditions.

## Interactions of Genetic Variants and Epigenetic Patterns for Complex Disease Risk

A common SNP is defined as a single base change in a DNA sequence that occurs among a significant proportion (≥1%) of a population (Lockwood et al., 2014). SNPs may reside in coding regions or non-coding regions. Contrary to the common belief, it is estimated that about 90% of GWAS-associated genetic variants reside in non-coding regions (Welter et al., 2014; Farh et al., 2015). While variants in coding regions may confer disease risk through altered protein sequences and, therefore, altered protein functions, variants in non-coding regions (usually enhancers) contribute to disease susceptibility through changing gene transcription and non-coding RNAs (Hrdlickova et al., 2014). These genetic variants might engage in crosstalk with sequence-specific transcription factors and epigenetic patterns (including DNA methylation) (McVicker et al., 2013), thereby impacting the transcription of genes locally or remotely.

### Genetic Variants in Coding Regions for Disease Susceptibility via Altering Epigenetic Patterns

**Figure 2:**
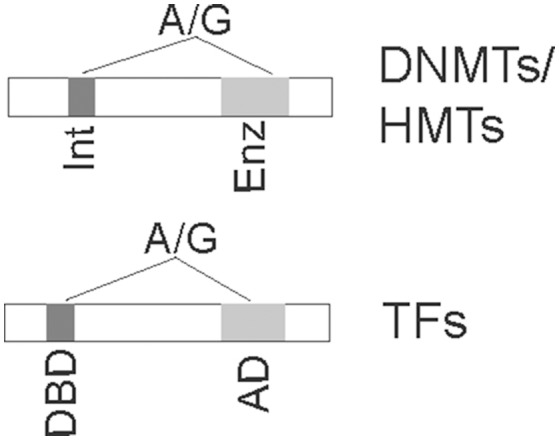
SNPs in critical domains of coding regions of epigenetic enzymes (**Top**) or transcription factors (**Bottom**) for disease risk are depicted. SNPs illustrated in the top panel may change the enzymatic activities if in enzymatic domain (Enz) or may change the interacting domain (Int) for interaction with other proteins, resulting in altered epigenetic marks. SNPs in the bottom panel will either abolish the DNA-binding domain (DBD) to target consensus sequences or impair activation domain (AD) for recruitment of epigenetic enzymes, thereby altering epigenetic patterns at target genes.

There are at least two scenarios, including SNPs within epigenetic enzymes and SNPs in TFs (Figure 2), that lead to the altered epigenetic patterns for increased disease risk. SNPs within epigenetic enzymes (including DNMTs and histone modifying enzymes) may potentially alter their functions, thereby subsequently changing epigenetic patterns in a genome-wide fashion. SNPs in TFs may impact the function of TFs' binding to DNA or its recruitment of epigenetic enzymes, thereby changing epigenetic patterns indirectly (Khandanpour et al., 2012).

SNPs in DNA methyltransferases have been reported in association with complex diseases. For example, polymorphisms in three DNMTs with methylation activity (DNMT1, DNMT3A, and DNMT3B) and in one DNMT without methylation activity (DNMT3L) are associated with an increased risk of schizophrenia (Saradalekshmi et al., 2014). Though many SNPs reside inside non-coding regions, at least one of them (rs2228611, within DNMT1 exon) has been found to be significantly associated with schizophrenia at genotypic and allelic levels in a South Indian population. SNPs in DNMT1 (exonic rs16999593) and DNMT3A (intronic rs1550117) may contribute to the gastric cancer risk, according to a recent meta-analysis (Li et al., 2016). Polymorphism rs1550117 of DNMT3A has been shown in association with the late-onset Alzheimer's disease. Specifically, patients with an AA genotype showed a 2.08-fold risk when compared to patients with a GG genotype (Ling et al., 2016). When investigating the imprinting disorder Beckwith-Wiedemann syndrome, Dagar and colleagues screened variants within the DNMT1 coding region and identified three patients (out of 53 examined) who contained three rare missense variants: rs138841970: C>T, rs150331990: A>G, and rs757460628: G>A; encoding NP_001124295 p.Arg136Cys, p.His1118Arg, and p.Arg1223His, respectively (Dagar et al., 2018). Using the DNMT1 binding as a surrogate for DNMT1 enzymatic activity, GFP-tagged DNMT1 fusion proteins with site-directed mutation show a reduced binding affinity (40-70%) of variants compared to that of the wild-type DNMT1. While it would be more informative to validate the expected DNA demethylation with bisulfite sequencing in three patients, the report at least provides a reasonable support for an association between variants in DNMT1 and increased disease risk (Dagar et al., 2018).

SNPs in histone-modifying enzymes and/or cofactors are also associated with complex disease susceptibility. GWAS from the GABRIEL Consortium (a multidisciplinary study of genetic and environmental causes of asthma) identified significant SNPs within histone-modifying enzymes in asthmatic patients, including histone deacetylases (HDAC4, HDAC7, HDAC9) and H3 lysine 36 demethylases (KDM4C, KDM2A) (Moffatt et al., 2010; Kidd et al., 2016). A focused summary of SNPs and mutations within histone lysine methyltransferases (KMTs) and histone lysine demethylases (KDMs) (Van Rechem and Whetstine, 2014) listed a few SNPs in coding regions of KMTs and KDMs in association with diseases. In contrast, the less frequent mutations or the deletion of KMTs and KDMs seem to be associated with more diseases. Although the variations in coding regions are expected to affect protein stability, folding, ligand-binding, and/or post-translational modification, it remains unclear how these SNPs impact the function of DNMTs and histone-modifying enzymes.

SNPs within coding regions of DNA-binding TFs can also potentially impact epigenetic patterns (Figure 2). These variants have an indirect effect, compared to the direct effect of SNP-altering epigenetic enzymes. The human growth factor independence 1 (GFI1), a DNA-binding transcription repressor, is important for hematopoietic stem cell and B and T cell differentiation. About 3 to 7% of white subjects contain GFI136N, which impairs binding to target genes such as *HOXA9*, resulting in elevated histone H3K4me2 signals and thereby promoting gene transcription. Compared with the GFI136S genotype, people with GFI136N have an increased risk (60%) for acute myeloid leukemia (Khandanpour et al., 2012). In one early cited report, identified common SNPs that have a potential impact on DNA binding of zinc finger TFs are unlikely to alter gene transcription in trans (Lockwood et al., 2014). However, Lockwood et al.'s results also suggest that large-scale analyses might offer a different conclusion, as in the case of GFI1.

### Genetic Variants in Non-coding Regions for Complex Disease Risk via Crosstalk With DNA Methylation Status and/or TF Binding for Shaping Chromatin Structure

**Figure 3:**
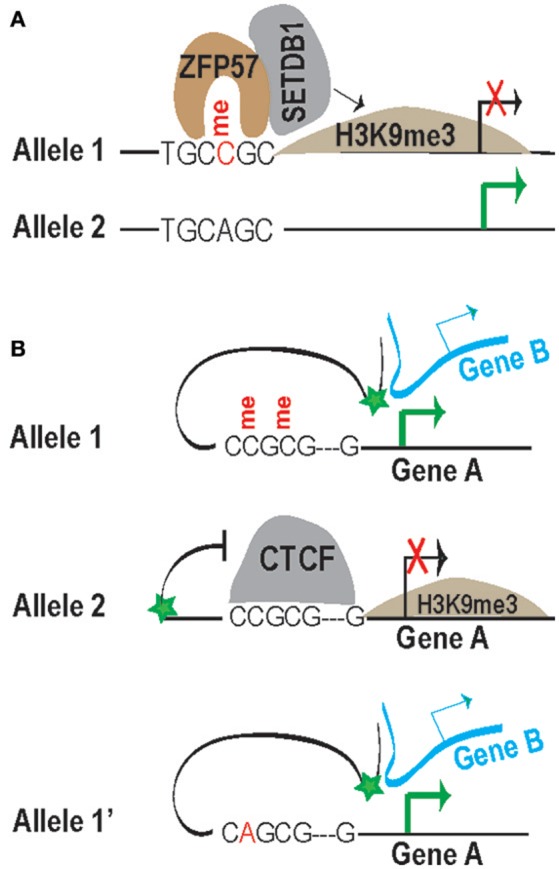
Illustration of methyl-CpG-dependent recruitment of ZFP57 **(A)** and methyl-CpG-sensitive binding of CTCF **(B)** for control of gene transcription. **(A)** CpG methylation within the motif is required for ZFP57 binding and subsequent recruitment of SETDB1 for the establishment of H3K9me3 locally. An SNP change of C to A abolishes ZFP57 binding. Without ZFP57/SETDB1-mediated repressive H3K9me3, genes in this allele 2 will be expressed. **(B)** In allele 1, the methylated CTCF motif prevents CTCF binding, thereby allowing upstream enhancers (green star) to access the transcription start sites (TSSs) for activation of gene A from the same chromosome and of gene B from a different chromosome. In allele 2, CTCF binds to the unmethylated motif, preventing enhancers to access the TSS of gene A for gene activation. In allele 1′, the SNP A replacing C will affect the binding of CTCF; therefore, allele 1′ will have a similar consequence to allele 1. Note that the enhancer may act to a different gene, such as gene such as gene B (top and bottom panel).

As aforementioned, about 90% of GWAS-identified genetic variants reside within the non-coding regions (Farh et al., 2015). SNPs in non-coding regions may impact gene function via several mechanisms. Intronic SNPs may affect splicing and/or mRNA stability, and studies have shown that an intronic SNP (rs910083-C) within *DNMT3B* is associated with an increased risk of nicotine dependence and squamous cell lung carcinoma. Though the mechanism is yet unclear, this SNP is associated with hypermethylation of about 252 bp upstream of the *DNMT3B* gene (Hancock et al., 2017). SNPs affecting the TFs' binding sites can predispose disease susceptibility by impacting the associated TFs' regulatory pathways (Figure 3), partially because SNPs at conserved residues within the consensus sequences for TFs can decrease or even abolish the binding of these TFs or because SNPs generate a new binding site for TFs. For example, the SNP A>C within the *Hcn2* locus for an allele-specific metal responsive element of metal regulatory transcription factor 1 (MTF1) (Martos et al., 2017). Hence, SNPs in this category could alter merely the DNA sequence, or, if they alter or generate CpG dinucleotides, they could simultaneously alter both the consensus sequence and its DNA methylation.

Accumulating evidence suggests that GWAS-identified and disease risk-associated genetic variants may largely confer their impacts by changing the expression of neighboring genes (Kilpinen et al., 2013; McVicker et al., 2013; Soldner et al., 2016; Allen et al., 2017; Gallagher et al., 2017; Li X. et al., 2017). Such changes may act through variant-disrupted motifs that eliminate or introduce the TF binding as described above. In our opinion, two TFs—ZFP57 and CTCF—are particularly intriguing to work with because of their known potential to impact chromatin in a global fashion (Ong and Corces, 2014; Riso et al., 2016). Both are DNA-binding TFs, and polymorphisms at different residues within their binding motifs seem to carry different weight with TF binding. In addition, these two TFs represent two distinct classes of TFs in terms of the impact of methylation status on their recruitments. That is, methylation is required for the binding of ZFP57 (Quenneville et al., 2011), while methylation prevents the binding of CTCF (Bell and Felsenfeld, 2000).

#### Methylation-Sensitive CTCF

##### CTCF biology

The insulator CTCF is an intriguing TF with potential roles for altering chromatin in a genome-wide fashion (Phillips and Corces, 2009; Ong and Corces, 2014). CTCF may also serve as a pioneer TF to shape local chromatin for recruiting additional TFs to their target sites (Tehranchi et al., 2016). In contrast to ZFP57, which is dependent upon methylation for binding, hypomethylation of the consensus sequence CCGCGNGGNGGCAG is critical for CTCF binding (Bell and Felsenfeld, 2000; Kim et al., 2007; Cuddapah et al., 2009). One essential role of CTCF is to block the spread of chromatin structure to the opposite side of a CTCF binding site (barrier) or to block the enhancer activity toward a promoter (Cuddapah et al., 2009; Phillips and Corces, 2009). The prototypical role of insulation has been revealed by investigation of CTCF at the imprinted *H19*/*Igf2* locus (Bell and Felsenfeld, 2000). *H19* and *Igf2* are located on the opposite side of CTCF-bound regions and there are a few enhancers downstream of the *H19* gene. CTCF binding motifs show allelic methylation, thereby dictating allelic CTCF binding. CTCF cannot bind to the methylated motifs of the paternal allele, allowing enhancers to access the paternal *Igf2* gene for activation. In contrast, CTCF binds to the unmethylated motifs of the maternal allele, thereby blocking enhancer access of *Igf2*, but allowing access of *H19* for stimulation. Experimental mutations to abolish CTCF binding affect imprinted expression within the locus (Singh et al., 2012).

CTCF's insulation is not limited to imprinted loci and is expected to act in a three dimensional fashion, presumably because enhancers can loop with multiple loci to regulate genes on the same or even different chromosome(s) (Spilianakis et al., 2005). Figure 3B presents a simplified cartoon to show the enhancer-blocking activity of CTCF in crosstalk with SNPs. In Allele 1, allelically methylated CpG sites prevent CTCF binding, enabling the enhancer to interact with gene A from the same chromosome and gene B from a different chromosome for stimulation. In Allele 2, the unmethylated motif allows CTCF insulation, thereby blocking the enhancer's access to stimulate gene A and, hence, keeping gene A silenced. A SNP change from C to A will potentially alter the conserved residues (illustrated in Allele 1′) and perhaps also the methylation status, preventing, or enabling CTCF insulation. Depending on the “conserveness” affected, the SNPs or mutations may reduce or even completely abolish CTCF binding to its motifs (Li W. et al., 2017), eventually affecting its insulation effect.

##### CTCF, genetic variation, and gene expression changes

Having briefly summarized CTCF insulation, we then did an in-depth examination of genetic variants within CTCF-binding motifs and their crosstalk with ABTF and ASMs in terms of impact on human health. Given the diverse functions of CTCF in transcription, imprinting, and X-chromosome inactivation (Phillips and Corces, 2009; Ong and Corces, 2014), it is not surprising that investigations begin to reveal the associations of these genetic variants with diseases or complex traits (Table 1) by impacting the allelic binding of CTCF. Among many reports (Table 1), several thorough investigations have presented their detailed insights of CTCF's allelic binding through long-range chromatin interactions that affect gene expression and, therefore, disease risks. Like ZFP57, CTCF's insulation or regulation of long-range chromatin interaction is not limited to ICRs of imprinted loci, but also applies to regular genomic regions (exemplified below).

**Table 1:** The crosstalk among CTCF, genetic variants, epigenetic variation in complex diseases or traits. (N/A, not available).

**Complex traits or diseases**	**Loci**	**Mechanism**			**Key results and/or model suggested**	**References**
		**Genetic variants**	**Epigenetic variation**	**ABTF**		
Asthma	ORMDL3	Yes	Yes	Yes	A SNP (rs4065275) in an enhancer within the 1st intron of *ORMDL3* promotes CTCF binding, whereas another SNP (rs12936231) downstream of the enhancer impairs CTCF binding. SNPs therefore alters three-dimentional organization in the asthma-risk allele to facilitate the expression of ORMDL3, which inhibits IL-2 production.	Schmiedel et al., 2016; Bérubé et al., 2017
Birth weight	H19/IGF2	Yes	Yes	N/A	A SNP rs10732516 A/G within the sixth binding motif of CTCF within the ICR results in allele-specific demethylation in paternal allele of rs10732516 paternal A/maternal G genotype. Allelic binding of CTCF is expected.	Marjonen et al., 2018
Cerebellum weight (CW)	H19/IGF2	Yes	Yes	Yes	DNA methylation at CTCF-binding site 3 within ICR explains ~25% of the CW variation; Genetic variation of the ICR in strong association with CW in a parental-origin dependent fashion.	Pidsley et al., 2012
Dementia	TMEM106B	Yes	Yes	Yes	The risk allele of rs1990620 increases the recruitment of CTCF, thereby leading to haplotype-specific effects on three-dimentional chromatin interactions and thus increased *TMEM106B* expression. The latter increases cytotoxicity for risk of neurodegeneration.	Gallagher et al., 2017
Influenza	IFITM3	Yes	Yes	Yes	A SNP (rs34481144) in the 5′ UTR of antiviral IFITM3 gene renders the risk allele with lower TF IRF3 binding but higher CTCF binding, thereby altering expression correlations among *IFITM3*-neighboring genes. The risk allele also disrupts a CpG site that is under differential methylation in CD8+ T cell subsets.	Allen et al., 2017
Lung cancer	DAGLA	Yes	N/A	N/A	A SNP within CTCF binding site inside an intron of *DAGLA* was significantly associated with increased risk of lung cancer	Dai et al., 2015
Lynch syndrome	MLH1	Yes			A SNP rs143969848 (G>A) within CTCF motif, part of an enhancer and also upstream of *MLH1* transcription start site, disrupts enhancing activity and MLH1 expression.	Liu et al., 2018
Mental illness	3p22 (TRANK1)	Yes		Yes	The risk allele of SNP rs9834970 shows lower baseline expression of *TRANK1* that may further alter genes important for neurodevelopment/differentiation. While the role of rs9834970 unknown, a nearby SNP rs906482 alters CTCF binding and the allele with increased CTCF binding is the risk allele of rs9834970.	Jiang et al., 2018
Osteoporosis	SOST	Yes		Yes	Four SNPs within the locus of *SOST* (negative regulator of bone formation and positive regulator of bone resorption). Among them, the SNP rs1230399 shows FOXA1 binding activity, resulting a T allele-specific activation; the SNP rs1107748 renders C allele transcriptional enhancer activity through a CTCF binding site; Variant rs75901553 C >T abolishes the binding site of miR-98-5p that is negative responsive to parathyroid hormone.	Ye et al., 2018
Osteoporosis	1p36.12 (LINC00339)	Yes		Yes	A SNP rs6426749 functions as a distal allele-specific enhancer stimulating the expression of a lncRNA	Chen et al., 2018
Type 2 diabetes	TF binding Sites	Yes	N/A	N/A	SNPs within motifs of CTCF, EP300, FOXA1/2, HNF4A, and TCF7L2 are associated with T2D from computational analyses	Cheng et al., 2017

For example, severe human influenza was reported in association with SNP rs34481144 A/G in the 5′ UTR of a regular *IFITM3* gene (not imprinted) that encodes antiviral protein IFITM3 for inhibition of viral entry (Allen et al., 2017). IFITM3 in memory CD8^+^ T cells promotes the adaptive immunity for antiviral resistance. Functional studies have revealed that the risk allele A decreases the binding of interferon regulatory factor 3 (IRF3) but increases the binding of CTCF. The latter is expected to impact the expression of *IFITM3*-neighboring genes via its insulation activity. Authors also demonstrate that increased methylation at rs3448114 blocks the binding of CTCF, thereby enhancing the expression of *IFITM3*. These investigations exemplify our proposed “SNP intensifier” model; a tiny difference at one residue (A/G, with the former disrupting a CpG site) is intensified via changing allelic DNA methylation, thereby affecting CTCF affinity and altering the expression of neighboring genes for disease risk (Allen et al., 2017). While this line of study has been limited to *in vitro* assays and would be much improved with *in vivo* confirmation of CRISPR/cas9-altered SNP A/G, the presented results are agreeable and consistent with the model presented later in section Conclusion, Challenges, and Perspectives.

Another example is the investigation of birth weight of babies conceived through assisted reproductive technology (ART) that was related to the imprinted locus. In ART, fresh embryo transfer-derived newborns are associated with low birth weight, while newborns derived from frozen embryo transfer are associated with increased birth weight (Marjonen et al., 2018). The underlying mechanism remains largely unknown. ART is expected to increase imprinting defects (Eroglu and Layman, 2012); therefore, the *IGF2*/*H19* locus, with its role in normal placental and embryonic growth, was selected for characterization. As described above, the *IGF2*/*H19* locus is regulated by an ICR that bears seven CTCF-binding motifs. In placentas from women who have used ART, a SNP rs10732516 A/G within the sixth binding motif (CCGCGc/tGGNGGCAG or complementary strand CTGCCNCCa/gCGCGG) of CTCF results in allele-specific demethylation on the paternal allele of rs10732516 paternal A/maternal G genotype, but not on the paternal G/maternal A genotype (Marjonen et al., 2018). The SNP allele A would interrupt the first CpG site of the complementary strand sequence CTGCCNCCa/gCGCGG and is associated with hypomethylation. It seems that the first CpG site might carry more weight in controlling DNA methylation status than do the following two CpG sites. These investigations also lack the expression status of *H19* and *IGF2* among genotypes (A/A, A/G, and G/G) for further mechanistic analysis. Fresh embryo transfer-derived newborns with the G/G genotype have shown an increased birth weight and larger head circumference when compared to the parameters of the A/A genotype (Marjonen et al., 2018).

The same group above has also examined SNP rs10732516 at the *IGF2*/*H19* locus for the consequences of prenatal alcohol exposure, which is known to affect development of the fetal nervous system and to restrict fetal head growth (Treit et al., 2016). Investigators found that alcohol exposure decreases the hypermethylation of the paternal allele of rs10732516 paternal A/maternal G genotype in placentas (Marjonen et al., 2017).

Additional reports listed in Table 1, including rs1990620 within the *TMEM106B* locus in association with neurodegeneration, support the “SNP intensifier” working model (presented later) (Gallagher et al., 2017). Lastly, CTCF is linked with intellectual disability (Bastaki et al., 2017; Hori et al., 2017). Altogether, a change of one residue (for example, C to A in Figure 3) may abolish or introduce the allelic recruitment of CTCF (and its associated insulation or long-range chromatin interactions), thereby causing a change from biallelic to monoallelic (or vice versa) expression of genes, and thus the copy numbers of the mRNA of related genes are altered.

##### Additional methylation-sensitive TFs

Many additional TFs—including bHLH, bZIP, and ETS family members—are also inhibited by methyl-CpG (Yin et al., 2017). Therefore, any SNPs that change these TFs' consensus motifs and/or result in methyl-CpG (e.g., “AG” becoming methylation-prone “CG”) have the potential to alter the binding of these TFs and, subsequently, to alter these TFs-controlled genes. Future investigations in these similar TFs will certainly be promising.

#### Methylation-Dependent ZFP57

The DNA-binding transcription factor ZFP57 only binds to the methylated TGCCGC hexanucleotide and, subsequently, recruits cofactor KAP1 and DNMTs, as well as SETDB1 (Quenneville et al., 2011). The recruited SETDB1, as a histone-modifying enzyme, is expected to establish H3K9me3 heterochromatin mark around ZFP57-targeted sites (Figure 3A) (Anvar et al., 2016). In addition, polymorphisms at CpG dinucleotides will not only change the consensus residue, but also change the methylation status (namely CpG-SNP), which may impact TF binding more severely. The A replacing the C in Allele 2 will change the consensus residue and methylation status (Figure 3A). Indeed, when AT replaced the fourth and fifth residues CG, the resulting oligonucleotide (mut1) was shown to lose both the consensus sequence and its methylation status. When TGC was changed to GAG, the resulting oligonucleotide (mut2) only altered consensus sequences, but still maintained the methyl-CpG. Intriguingly, the mut1 probe did not compete as well as the mut2 probe, at least in electrophoresis shift assays (Quenneville et al., 2011), suggesting that the methylation status may be more critical than the consensus sequence for ZFP57 binding.

While the association of methylated TGCCGC in H3K9me3 patterns was initially investigated in imprinting control regions (ICRs) of imprinted loci (Quenneville et al., 2011), regular genomic regions are expected to have similar ZFP57-associated H3K9me3. In other words, so long as genomic loci contain the TGCCGC sequences, these loci are potentially subjected to ZFP57/SETDB1-regulated H3K9me3. Therefore, SNPs at ZFP57 motifs are expected to play a role beyond imprinted genes (Anvar et al., 2016). Because ZFP57-mediated H3K9me3 signal distribution seems to have a relatively long range, any alterations of TGCCGC sequences could potentially affect the transcription of multiple genes within the region.

**Figure 4:**
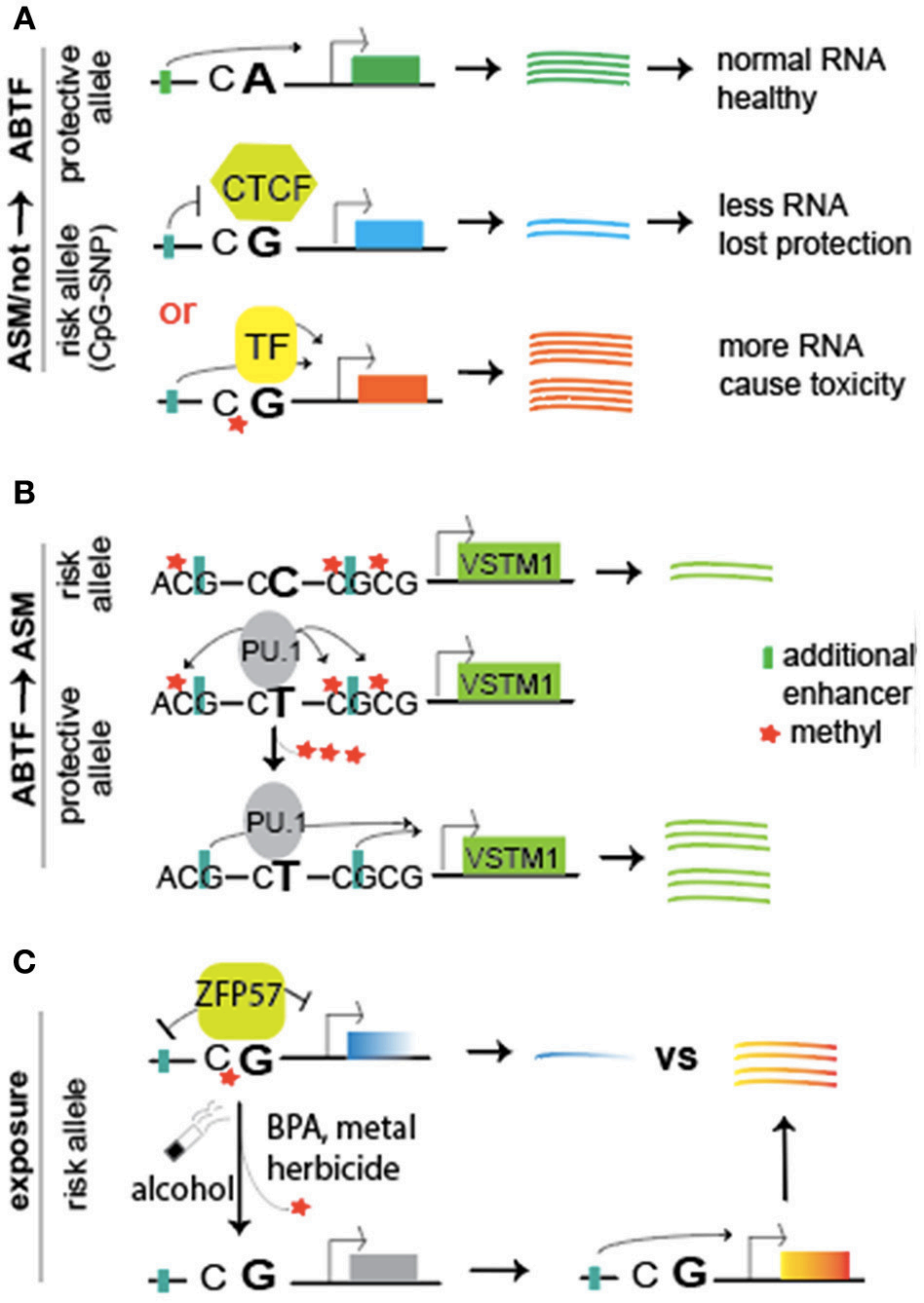
A working model of “SNP intensifier via allele-specific binding of TFs,” either having a crosstalk or not with allele-specific methylation. **(A)** Compared to the protective allele with CA dinucleotides (Top), the risk allele bears a CG site that can be maintained in unmethylated (Middle) or methylated (Bottom) status. The former may result in methylation-sensitive recruitment of transcription factors such as insulator CTCF, preventing an upstream enhancer to stimulate gene transcription for less RNA copies. Therefore, less RNA transcripts may be not good enough to provide necessary protective roles like the protective allele. In contrast, the methylated status may recruit methylation-dependent active TFs (or repressors) for stimulation of gene transcription, resulting in more RNA copies and thereby causing toxicity to the cells. Note the change from CA on protective allele to CG on risk allele, introducing a CpG-SNP. **(B)** Illustrate a model of SNP-resulted ABTF (e.g., protective T allele recruiting PU.1) that initiates the Tet-mediated demethylation of neighboring CG sites on the allele it binds (Kumar et al., 2017). Such demethylation may relieve the inhibitory role of methyl group on enhancers for stimulation of gene transcription. In contrast, the risk C allele does not recruit PU.1 for demethylation. **(C)** Introduce the crosstalk with exposure. The methyl-CpG site (e.g., within a binding motif of ZFP57) is sensitive to environmental exposure (alcohol, BPA, cigarette smoke, herbicides, and metals). The resulting demethylation eliminates methyl-CpG-dependent binding of TFs (such as ZFP57), thereby abolishing ZFP57′s repression of genes. The simulated expression from upstream enhancers leads to allelic imbalance for disease risk. In all cases **(A–C)**, the total RNA copies were altered, resulting in a disastrous dosage effect for disease risk.

The effect of SNPs within the ZFP57 motifs on disease risk remains to be determined in human populations. Our literature search was relatively futile in this aspect; however, a few reports indeed linked methylation QTLs to expression changes of ZFP57 in disease/traits, including post-traumatic stress disorder (Rutten et al., 2018), metabolic trait (Volkov et al., 2016), and psychosis (Rivollier et al., 2017). Compared to the extensive studies of SNPs within CTCF binding motifs for complex traits/diseases (which burgeoned only in the past 2 to 3 years) (Table 1), we expect more upcoming reports to show the effect of SNPs within ZFP57 binding motifs on disease risk because these SNPs seem to abnormally alter allelic expression of genes (Anvar et al., 2016). From F1 hybrid ES cells between C57BL/6 and Cast/EiJ, genetic variants with disruption of ZFP57 consensus motif and methylation status are linked to monoallelic expression of neighboring genes (Strogantsev et al., 2015). Note: Allelic increases or decreases of gene expression are key for CTCF-involved disease risk (Figure 4).

In summary, this section presents the mechanistic insights of SNP-resulted allele-specific methylation that facilitates the allelic binding of TFs such as ZFP57 (Figure 4A, bottom panel) and of SNP-resulted motifs (unmethylated) for insulator CTCF (Figure 4A, middle panel). In both cases, ABTFs play a key role in allelic regulation of gene transcript. In the following section, we will review SNP-resulted ABTF, thereby leading to the subsequent ASMs and potential ASE (Figure 4B).

## Genetic Variants in Shaping Allele-specific DNA Methylations (ASMs) and Potential Allele-specific Gene Expression (ASE) for Disease Risk

### Unexpected ASMs and ASE for Autosomal Genes in the Human or Mouse Genome

It is worth presenting genetic variants in shaping ASMs (separately from the above section Interactions of Genetic Variants and Epigenetic Patterns for Complex Disease Risk). In this section, we envision the underlying mechanism for ASMs and ASE. Traditionally, both ASM and ASE are considered as the phenomena of genomic imprinting and X chromosome inactivation (XCI). However, more recent reports have started to unveil unexpected allelic asymmetries of many non-imprinted autosomal genes in the mammalian genome (Klengel et al., 2013; Izzi et al., 2016).

Increasing evidence shows that both the human and mouse genomes contain hundreds or even thousands of ASMs (Cheung et al., 2017). Originally aimed to identify unknown imprinted genes, one study identified many unexpected ASMs from genomic loci outside of known imprinted regions in several human tissues (Kerkel et al., 2008). Further investigations demonstrate that these ASMs are tissue-specific and individual-specific, suggesting genetic background in affecting ASMs (Yang et al., 2010). Looking at two mouse strains (C57BL/6 and BALB/c) and their F1 hybrid offspring, investigators focused on 181 large genomic intervals with an approach based on methyl-CpG immunoprecipitation and locus-wide tilling arrays, identifying several hundreds of differentially methylated regions and strain-specific methylation patterns controlled by *cis*-acting polymorphisms (Schilling et al., 2009). Regarding the identification of unknown imprinted loci in the mouse genome, our group has developed a computational approach based on the feature of monoallelic hyper- or hypomethylation at ICRs to scan the base-resolution DNA methylomes from the mouse ES cell J1 line (Li et al., 2015; Martos et al., 2017) that identified more than 2,000 regions showing bimodal methylation patterns. These regions are potentially associated with monoallelic methylation. In the subsequent validation, we generated four independent hybrid ES lines (from a reciprocal cross between 129S1/SvlmJ and Cast/EiJ or between C57BL/6NJ and Cast/EiJ) and confirmed the ASM of the *Hcn2*/*Polrmt* locus—that is, within this locus, the Cast allele with a SNP C is always hypomethylated (i.e., independent of parental origin), whereas the 129 allele or the C57 allele with a SNP A is always hypermethylated. Intriguingly, the SNP C renders the Cast allele a new motif for metal regulator transcription factor MTF1 (Martos et al., 2017).

### Do Non-imprinted ASMs Control ASE?

The extent to which the non-imprinted ASMs control allelic gene expression, remains to be defined. Because imprinted ASMs control ASE, one would expect the similar control of ASMs for the allelic expression of genes on autosomes. Indeed, reports support that ASMs are at least one of the factors to cause allelic imbalance of gene expression.

1. **Genetic variants-resulting demethylation of one allele for ASM and ASE**. This class of variant regulation is initially featured by SNP-facilitated ABTF, which initiates allelic demethylation for ASM and ASE (Figure 4B). In other words, ABTF leads to ASM. One example is an atopic dermatitis-associated SNP rs612529 T/C in the promoter of *VSTM1* that encodes SIRL-1. It turns out that the protective T allele facilitates the recruitment of transcription factors YY1 and PU.1 (Kumar et al., 2017). YY1 can either activate or repress gene transcription, depending on the context in which it binds (Gordon et al., 2006), whereas PU.1 seems to demethylate its target genes through Tet2 demethylation (de la Rica et al., 2013). Indeed, the neighboring CpG sites of the PU.1 binding site on the protective T allele, as compared to those of the risk C allele, are hypomethylated in monocytes, thereby leading to allelic upregulation of gene transcripts. The risk C allele does not recruit PU.1, resulting in the low expression of SIRL-1 in monocytes. The latter leads to a higher risk for manifestation of an inflammatory skin disease (Kumar et al., 2017). Another example is the allele-specific demethylation of long-range enhancers of the *FKBP5* (FK506 binding protein 5) gene, which increases the susceptibility of developing stress-related psychiatric disorders in adulthood (Klengel et al., 2013). FKBP5 is an important regulator of the glucocorticoid receptor complex that is involved in the stress hormone system. Exposure to early childhood abuse is associated with demethylation of the enhancer on the risk allele (with the AA genotype), bringing the enhancer through long-range interaction to transcription machinery for allelically increased transcript of *FKBP5*. The latter results in a long-term malfunction of the stress hormone system and a genome-wide impact on the function of immune cells (Klengel et al., 2013). The third example is growth differentiation factor 5 (GDF5), a ligand of the TGF-beta superfamily of proteins, essential for normal skeletal development. In the 5′ UTR of the *GDF5* gene, a C-to-T SNP rs144383 is a risk factor for osteoarthritis of the knee. Methylation of a highly conserved CpG site (4 bp upstream of rs144383 and part of SP1 and SP3 motif) affects allele-specific binding of repressor SP1 and SP3, thereby attenuating the repressive effect of SP1 and SP3 proteins and resulting in allelic expression of *GDF5* (Reynard et al., 2014). This ABTF of SP1 and SP3 may exemplify how the above mentioned MTF1 predisposed the hypomethylation of Cast allele within the *Hcn2* locus in our studies. Presumably, this MTF1 predisposes the Cast allele for Tet proteins for demethylation; however, the extent to which the ABTF predisposes allelic hypomethylation remains to be determined (Martos et al., 2017). Working on chromosome 21 in leukocytes from healthy individuals, investigations also show that the genetic variation among individuals affects ASMs, thereby leading to ASE (Zhang et al., 2009). In acute lymphoblastic leukemia, ASE for 470 SNPs within 400 genes was detected. The level of ASE varies from a 1.4-fold overexpression of one allele to strictly monoallelic expression. Further investigation suggests that ASE is associated with promoter CpG site methylation (Milani et al., 2009).
2. **Genetic variants-resulting methylation of one allele for ASM and ASE**. In contrast to the demethylation of one allele for ASMs mentioned above, genetic variant-related methylation of one allele can also lead to ASMs and potential ASE. For example, the SNP rs12041331 has been linked to cardiovascular disease and platelet reactivity (Izzi et al., 2016). The major G allele of rs12041331 (leading to a CpG-SNP within the intron of the *PEAR1* locus) is linked to a higher transcript level than the minor A allele in endothelial cells and platelets. The resulting CpG site of GG carriers is fully methylated in leukocytes. Intriguingly, this methylated CpG site of the G allele recruits more nuclear proteins than does the unmethylated A allele. However, the authors did not characterize these nuclear proteins (Izzi et al., 2016); otherwise, their insights would be clearer. Based on our summary above, it is reasonable to expect a similar binding of TFs like ZFP57 to the methylated CpG site. That is, methylation-dependent recruitment of c-Jun and/or ATF3 (resulted due to CpG-SNP on the G allele) may understand the higher *PEAR1* expression and risk. More recent reports linking ASMs to disease or complex traits include the SNP rs174537-regulated ASM at the *FADS* gene locus for long-chain polyunsaturated fatty acid biosynthesis (Rahbar et al., 2018) and ASMs of susceptibility genes for inflammatory bowel disease (Chiba et al., 2018).

### Is Dosage Effect the Key for Disease Risk?

Imprinting disorders have been linked to abnormal biallelic expression or biallelic silencing of imprinted genes in contrast to monoallelic expression (Bartolomei, 2009; Weksberg, 2010). The dosage effect is certainly key for imprinting disorders. As for non-imprinted loci with ASMs and/or ASE, we expect a similar role. Allelic imbalance—a hallmark of cancer—has been known to contribute to cancers for many years. For example, monoallelic expression of cancer-related genes, including *TP53* and *IDH1*, seems to be in association with tumor aggressiveness and progression (Walker et al., 2012). The ASE of *BRCA1* and, to a lesser extent, of *BRCA2*, contributes to an increased risk for breast cancer (Chen et al., 2008). ASE is also observed in acute lymphoblastic leukemia, as described above (Milani et al., 2009). Our thorough examinations including insulator CTCF-involved diseases/traits (examples in Table 1) and other TFs-mediated traits/diseases (described above) demonstrate the causative increase or decrease of gene transcripts of one allele. In the authors' opinion, this is a dosage effect, like imprinting disorders for complex traits or diseases.

## Vulnerability of Genetic Variant-influenced ASMs and/or ASE in Response to Environmental Factors for Disease Risk

The extent to which these genetic variants-influenced ASMs and ASE are vulnerable to environmental factors remains to be determined. However, we may learn from previous investigations on imprinted ASMs, as both imprinted ASMs and genetic variants-influenced (or non-imprinted) ASMs share the feature of mono-allelicity. Since there is no backup from the complementary allele, the mono-allelicity of methylation, presumably, is the cause for vulnerability. It is, therefore, reasonable to expect that ASMs (both imprinted and non-imprinted) are vulnerable to exposure.

The imprinted ASMs at ICRs, the key for imprinted gene expression, are known to be vulnerable to environmental exposures, including BPA and cadmium, as they can alter the allelic expression of imprinted genes and, consequently, increase disease susceptibility (Heijmans et al., 2008; Susiarjo et al., 2013; Van de Pette et al., 2017; Cowley et al., 2018). Indeed, in response to cadmium exposure, ASMs at ICRs do show a higher sensitivity to cadmium when compared to other loci in newborn cord blood and maternal blood (Cowley et al., 2018). As for alcohol use disorder, prenatal alcohol exposure alters one ASM of the *H19*/*Igf2* locus, thereby causing about a 1.5-fold decrease of *Igf2* transcripts (Downing et al., 2011). The ASMs of imprinted loci, including *Dio3*, and *H19*/*Igf2*, are susceptible to fetal alcohol exposure, thereby changing the allelic expression of *Dio3* and *Igf2* (Haycock and Ramsay, 2009; Tunc-Ozcan et al., 2016, 2018). Lastly, calorie restriction can also alter imprinted *Igf2* expression in a sex-dependent manner. In rats, moderate calorie restriction during gestational days 8 through 21 of Sprague-Dawley dams (F0) increases the adult hippocampal *Igf2* transcripts in F1 females, and in these F1 females-produced (in cross with naïve male Brown Norway) F2 offspring (Harper et al., 2014). Although it was not fully investigated as to whether calorie restriction altered ASM within the *H19*/*Igf2* locus for this intergenerational increase of *Igf2* in female offspring, similar treatment with maternal vitamin D depletion has suggested changes in DNA methylation (Xue et al., 2016).

With the established vulnerability of imprinted ASMs to exposure, the non-imprinted ASMs are expected to behave similarly. As exemplified above, the risk allele (AA carrier) of the *FKBP5* locus is indeed vulnerable to child abuse exposure (Klengel et al., 2013). With social or mental stress, the oxytocin receptor gene (*OXTR*) SNP rs53576 (G-A) is expected to be associated with social behavior. Investigations demonstrate that prenatal mental stress exposure is linked to child autistic traits, but not related to *OXTR* methylation across the rs53575 G allele homozygous children or A allele holder (Rijlaarsdam et al., 2017). Though not clear about the allelic sensitivity to mental stress from the investigation, the *OXTR* methylation levels were positively in association with social problems for the G allele homozygous children (but not the A allele carriers). Using five human cell types for 50 treatments, Luca and colleagues identified 1,455 genes with ASE and 215 genes with gene-by-environment (GxE) interactions (Moyerbrailean et al., 2016). More importantly, exposure-perturbed genes showed a 7-fold increased odds of being reported in GWAS. Almost half of 215 genes showing GxE interactions are associated with complex traits, as revealed by GWAS. These results are consistent with the idea that genes with ASE are vulnerable to exposure. Additional evidence regarding alcohol use disorder also supports this idea. In rats, drinking alcohol affects the ASE of about 300 genes, as reported recently from Zhou and colleagues (Lo et al., 2018). This affection is expected through the crosstalk among genetic variants, epigenetic patterns, and alcohol exposure.

## Conclusion, Challenges, and Perspectives

Accumulating evidence supports that the GWAS-identified variants in non-coding regions are implicated in complex human diseases or traits, an implication that involves an extensive crosstalk among SNPs, ASMs, and ABTFs for impacting ASE (Figures 4A,B), as well as environmental exposure-altering ASMs and ABTFs (Figure 4C), thereby rendering a person more susceptible to diverse diseases. Exposure to environmental factors (bisphenol A, metal, herbicides), alcohol, and cigarette smoke has been demonstrated to affect chromatin structure (DNA methylation and histone modifications), thereby potentially altering ASMs and ABTFs (Cowley et al., 2018; Meehan et al., 2018; Pathak and Feil, 2018; Strakovsky and Schantz, 2018; Zhu et al., 2018). With more investigations of the crosstalk of SNP-TFs-allelic expression changes and of SNP-insulator CTCF-allelic expression changes, which have burgeoned only in the past 2 years (Table 1), it becomes clearer that the signal of a small change at one residue (i.e., SNP) is intensified by introducing or abolishing allelic DNA methylation, thereby impacting the allelic recruitment or abolishment of TFs. The allele-specificity of TFs eventually increases or decreases transcript copies from one allele, and the resulting changes of dosage seem to be the key for disease risk (Figure 4). We thus coin this working model as the “SNP intensifier.”

A future challenge is to provide more evidence to elucidate this mechanism of variants contributing to human disease susceptibility and inter-individual phenotypic differences. In regard to the elucidation, several roadblocks must first be cleared and a better standard must be established, including the quick characterization of all GWAS-associated genetic variants in affecting epigenetic patterns and gene transcription, and the conclusive determination of each variant's role in complex human disease susceptibility. More specifically, we should seek to identify all SNPs-resulted ASMs or methylation QTLs in a cost-effective and high throughput manner, characterize the chronic effect of slightly increased RNA transcripts of a gene for disease risk, and integrate layers of information from variants, ASMs/ASE, and exposure for deeper mechanistic insights.

The community certainly needs an integrated system for biological analyses of data from layers of genetic variants, epigenetic patterns, and expression QTL (eQTL). Intriguingly, a summary data-based Mendelian Randomization (SMR) method was recently developed (Zhu et al., 2016). The method of SMR borrows the concept of Mendelian Randomization (MR) analysis, which uses a genetic variant (e.g., a SNP) as an instrumental variable to assess the putative causative effect of an exposure (e.g., gene expression level) on an outcome (e.g., diseased phenotype). Because the variance in a phenotype explained by a single genetic variant, or the expression change of a single gene, is likely to be very small, a limitation of MR analysis is the requirement of an extremely large sample size. To overcome this limitation, Zhu et al. used the summary-level data (for instance, effect sizes or test statistics) available from the very large-scale GWAS and eQTL studies (Zhu et al., 2016). With SMR analyses, investigators integrated summary-level GWAS data on up to 339,224 individuals and eQTL data on 5,311 individuals for the identification of 126 genes in association with five human complex traits. Out of these 126 genes, *TRAF1* and *ANKRD55* were found to be associated with rheumatoid arthritis, and *SNX19* and *NMRAL1* were found to be associated with schizophrenia. Using the SMR approach, an independent group identified DNA methylation sites in association with GWAS-identified variants for multiple complex traits out of more than 40 traits examined (Hannon et al., 2017) and showed the potential role of genetic variants in the *RNASET2* locus in association with eQTL and mQTL (methylation quantitative trait locus) for Crohn's disease. Similarly, the group that first reported the SMR method also integrated the summary-level data of mQTL with that of GWAS and eQTL (Wu et al., 2018) and revealed pleiotropic associations between 7,858 DNA methylation sites and 2,733 genes, which can be regarded as a map of the methylome to the transcriptome. Further analyses identified 149 DNA methylation sites and 66 genes showing pleiotropic associations with 12 complex traits. Wu et al. hypothesize a mechanism whereby a genetic variant impacts complex disease by way of genetic modulation of transcription through DNA methylation.

Characterization of genetic variant-influenced ASMs remains a challenge as well. Not surprisingly, these ASMs show tissue specificity (Do et al., 2016; Marzi et al., 2016). Therefore, the cost-effective identification of all ASMs in different tissues is needed. With tissue specificity, methylation status of CpGs within a given ASM can be changed during cell development, and differentiation. For example, our data demonstrate that HCN2 ASM seems to exist in embryonic stem cells, but that CpG sites of unmethylated allele were methylated in differentiated neural progenitor cells and neurons (Martos et al., 2017). In addition, the characterization of vulnerability of each ASM, from different tissues to different environmental stresses, is also critical for understanding the complex human diseases. Lastly, the current method to analyze DNA methylation for large cohort studies relies on an array-based approach that is limited to low resolution, and there is an urgent need for an alternative sequencing-based method for efficient screening of hundreds or thousands of samples.

Due to the accompanied ethnic issues of human samples, animal studies are required to solve the mystery of the detailed mechanistic insights. For example, animal studies are needed for exploration of the effect of chronic and even subtle changes of one allele. F1 hybrid mice, between two strains of rat or mouse, have many more SNPs (for example, up to 25 million SNPs between mouse strains 129 and Cast) for insights (Schilling et al., 2009; Lossie et al., 2014; Lo et al., 2016; Martos et al., 2017). The interactions between genetic variants and disease susceptibility may be tested via different crossing strategies in the Diversity Outbred mouse population and the Collaborative Cross inbred stains (French et al., 2018), another hot topic that will not be described in detail in this review.

While the mechanistic insights gained through the extensive hard work above have brought the community closer to our ultimate goal of finding opportunities for the intervention and prevention of human diseases, at least one investigation has shown encouraging results in this regard. Administration of thyroxin (T4) or metformin to neonatal rats after fetal alcohol exposure was shown to reverse the expression changes of *Dio3* and *Igf2* and to alleviate the fear memory deficit that was triggered by fetal alcohol exposure (Tunc-Ozcan et al., 2018). This reverse encourages the exploration of drugs for potential intervention.

Finally, the evidence from our own studies suggests the existence of ASMs that are independent of SNPs (Martos et al., 2017) in mouse embryonic stem cells that were generated from an inbred mouse strain 129 (i.e., no SNPs between two alleles). To understand the significance and mechanism of these random ASMs, we envision a similar mechanism to XCI and propose a hypothesis of regional “autosomal chromosome inactivation (ACI)” (Martos et al., 2017). However, the significance and impact of ACI on human health remains a mystery.

## Author Contributions

HW, DL, and ZW prepared the manuscript. HW and DL contribute significantly in terms of literature reading, summarizing, and presenting.

### Conflict of Interest Statement

ZW is a consultant of Shandong Bio-focus Gene-tech Co. Ltd. The remaining authors declare that the research was conducted in the absence of any commercial or financial relationships that could be construed as a potential conflict of interest.
